# Turnover Rates and Diet–Tissue Discrimination Factors of Nitrogen and Carbon Stable Isotopes in Seahorse *Hippocampus reidi* Juveniles Following a Laboratory Diet Shift

**DOI:** 10.3390/ani12101232

**Published:** 2022-05-10

**Authors:** Jorge Hernández-Urcera, Mario Davi Dias Carneiro, Miquel Planas

**Affiliations:** 1 Department of Ecology and Marine Resources, Institute of Marine Research (CSIC), 36208 Vigo, Spain; marioiddc@gmail.com; 2 Laboratório de Piscicultura Estuarina e Marinha, Instituto de Oceanografia, Universidade Federal do Rio Grande–FURG, Rio Grande 96201, RS, Brazil

**Keywords:** *Hippocampus*, seahorse, diet switch, stable isotopes, turnover, discrimination factor

## Abstract

**Simple Summary:**

The main aim of the present study was to ascertain the effect of two feeding schedules (including copepods and *Artemia* nauplii) on the early development and physiology of seahorse *Hippocampus reidi* juveniles. For that, we analyzed seahorse performance (growth and survival) and trophic patterns by means of stable isotopes. Our results highlight that the welfare and condition of juveniles were enhanced by extending the period of feeding on copepods up to day 10 after the male’s pouch release. The analysis of turnover rates for δ^13^C and δ^15^N revealed that switching copepods to *Artemia* nauplii at earlier developmental stages would reduce prey assimilation resulting in lower growth rates and survivals. The present study also provides for the diet–tissue discrimination factors for δ^13^C and δ^15^N in seahorse juveniles for the first time.

**Abstract:**

The initial development of seahorse juveniles is characterized by low digestion capabilities. Stable isotope analysis is an effective tool in studies of trophic food webs and animal feeding patterns. The present study provides new insights for the understanding of growth and food assimilation in early developing seahorses following a laboratory diet switch. The study was performed in the early life stages of the seahorse *Hippocampus reidi* by assessing the influence of diet shift on changes and turnovers in carbon (δ^13^C) and nitrogen (δ^15^N) stable isotope in juveniles. Newborn seahorses were fed for 60 days following two feeding schedules (A6 and A11) based initially on copepods *Acartia tonsa* and subsequently on *Artemia* nauplii (since days 6 and 11, respectively). After the prey shift, we determined δ^13^C and δ^15^N turnover rates as functions of change in either body mass (fitting model G) and days of development (fitting model D), contributions of metabolism and growth to those turnover rates, and diet–tissue discrimination factors. Survival, final dry weight, and final standard length for diet A11 were higher compared to diet A6. The shift from copepods to *Artemia* led to fast initial enrichments in δ^13^C and δ^15^N. Afterwards, the enrichment was gradually reduced until the isotopic equilibrium with the diet was reached. In most cases, both fitting models performed similarly. The isotopic analysis revealed that 100% of tissue turnover was attributed to growth in diet A11, whereas 19–25% was linked to metabolism in diet A6. Diet–tissue discrimination factors were estimated for the first time in seahorse juveniles, resulting in higher estimates for diet A11 (2.9 ± 0.7‰ for *δ*^13^C; 2.5 ± 0.2‰ for *δ*^15^N) than in diet A6 (1.8 ± 0.1‰ for *δ*^13^C; 1.9 ± 0.1‰ for *δ*^15^N). This study highlights the relevance of feeding on copepods and their effect on isotopic patterns and discrimination factors in seahorse juveniles after a dietary shift. Regarding the application of the results achieved in relation to the feeding schedules in the rearing of *H. reidi*, a long period of feeding on copepods during the first days of development is highly recommended.

## 1. Introduction

Dietary studies in fishes provide useful information to assess predator–prey relationships, competition, food intake, and food-web dynamics. Traditionally, they were based on gut content analysis, but stable isotope analysis (SIA) is an analytical tool that can be applied in dietary traceability studies and long-term food utilization by organisms [1,2,3,4,5]. The basic assumption of SIA is that the isotopic composition of an organism’s tissues reflects that of its diet [6] offset by a trophic discrimination factor [7].

The two most commonly measured stable isotope ratios are ^13^C/^12^C and ^15^N/^14^N; both ratios are usually higher in the consumer’s tissues compared to its diet because the lighter isotope (^12^C and ^14^N) is preferred in metabolic processes [8,9] and also because heavier isotopes are preferentially retained. Specifically, the stable-carbon isotope ratio (^13^C/^12^C) of tissues reflects the sources of organic carbon consumed, with little tissue fractionation [8]. Stable-nitrogen isotope ratios (^15^N/^14^N) increase with successive trophic levels [9], allowing estimates of the consumer’s trophic position [7,10,11,12].

Discrimination represents the difference between isotope values for diet and fully equilibrated consumer tissue [11]. Discrimination factors for carbon and nitrogen usually average 0–1‰ and 3–4‰ per trophic level, respectively [13], depending on the tissues/species considered [7,12]. However, the magnitude of this per trophic-step isotope fractionation (Δ^13^C or Δ^15^N) can be affected by other many factors such as diet quality, feeding ratio, nutritional stress, body size, age, physiological status, and excretory mechanisms [14,15,16,17,18,19,20].

Generally, stable isotope values are fitted to growth or time-based models [14,15,16,17,18,19,20]. Most laboratory diet-switch experiments [21,22,23,24,25,26,27] show that growth is the primary factor causing stable isotopic changes in fish following a diet shift. In the case of fish larvae, experimental stable isotope studies investigating the effects of a diet shift on stable isotope incorporation are scarce. These studies are relevant in identifying the diet preferences of larvae and juveniles, understanding nutrition needs, improving rearing techniques, and interpreting field stable isotope studies [28]. In addition, knowledge of species turnover and discrimination factors are important for the accurate interpretation of isotopic data.

*Hippocampus reidi* (Teleostei: Family Syngnathidae) is a tropical euryhaline seahorse, inhabiting estuarine areas along the Western Atlantic coast, mainly in Brazil [29,30,31]. It is one of the most significant seahorse species in the marine aquarium trade [32,33]. Previous studies in seahorse cultivation have demonstrated the limited digestive capability in early developing juveniles and the low digestion of *Artemia* nauplii compared to copepods [34,35,36]. Consequently, the initial feeding enhances the growth and survival of copepods. The present study was performed in the early developmental stages of *H. reidi* by assessing the influence of diet shift on isotopic changes (δ^13^C and δ^15^N) in juveniles fed on two different feeding schedules including copepods and *Artemia* nauplii. This experimental study aimed to determine for the first time in seahorses: (1) δ^13^C and δ^15^N turnover rates in juveniles as functions of change in body mass and time, (2) contributions of metabolism and growth to those turnover rates, and (3) diet–tissue discrimination factors.

## 2. Materials and Methods

### 2.1. Broodstock

Adult seahorses *Hippocampus reidi* Ginsburg, 1933 were maintained in ad hoc aquaria [37] at the Instituto de Investigaciones Marinas (IIM-CSIC) in Vigo (Spain) and fed twice daily on a diet consisting of long-time enriched adult *Artemia* sp. (EG, AF, MC450; Iberfrost, Tomiño, Spain; 40–70 *Artemia* seahorse^−1^ dose^−1^) [38] and frozen Mysidaceans *Neomysis* sp. (Ocean Nutrition, Essen, Belgium). When available, a single daily dose of wild-captured Mysidacea (15–20 *Leptomysis* sp. and/or *Siriella* sp.) was also provided. Seawater was maintained at a constant temperature within an annual temperature regime of 26 ± 0.5 °C. A natural-like photoperiod regime for the species was applied (16L:8D). Pumped seawater was filtered (5 µm), UV treated, and 10–15% was exchanged daily. Water quality was checked periodically for NO_2_, NO_3_, and NH_4_/NH_3_ content (0 mg L^−1^) using Sera Test Kits (Sera GmbH, Heinsberg, Germany). Salinity and pH levels were constantly maintained 38 ± 1 and 8.1 ± 0.1, respectively. Wastes and uneaten food were removed daily by siphoning the bottom of the aquaria.

### 2.2. Experimental Design

Seahorse juveniles were obtained from a batch released by one male held in captivity for 3 years. Immediately after the male’s pouch release, the juveniles were randomly transferred (3.3 juveniles L^−1^) into 4 (2 aquaria per treatment) 30 L aquaria [39]. The rearing system was illuminated by 20 W Power Glo lamps (Hagen, Montreal, Canada) under a 14 L:10 D photoperiod regime. Water temperature was adjusted to 26 °C. Total seawater volumes in the rearing system were replaced twice per hour by means of an external inflow (24 L h−1) of 20 μm filtered and UV-treated seawater. Aquaria were gently aerated in the upper part of the water column at a continuous flow rate of 700 mL min^−1^. Twice daily, wastes and feces were siphoned out, and dead seahorses were removed and counted.

Two feeding schedules (diets A6 and A11) were compared considering the following feeding conditions:

Diet A6

- First feeding (0 to 5 DAR): Two daily doses of cultivated copepods *Acartia tonsa* (1 copepod mL^−1^);
- *Artemia* feeding (6 to 60 DAR): Two daily doses of *Artemia* nauplii (1–2 *Artemia* mL^−1^).
- Diet A11
- First feeding (0 to 10 DAR): Two daily doses of cultivated copepods *Acartia tonsa* (1 copepod mL^−1^);
- *Artemia* feeding (11 to 60 DAR): Two daily doses of *Artemia* nauplii (1–2 *Artemia* mL^−1^).

The experimental feeding schedules were established considering previous results on the effect of diet in the early rearing of *H. reidi* juveniles [36]. In that study, we demonstrated an enhancement of seahorse juveniles with longer periods of feeding on copepods.

The copepods were cultivated in 700 L tanks at 26–27 °C and 38 salinity and fed every two days on microalgae *Rhodomonas lens* (10^3^ cells mL^−1^). Only copepods retained by a 125 μm mesh were offered to the seahorses. *Artemia* nauplii were obtained from cysts (AF, Inve, Spain) hatched at 28 °C for 20 h in 20 L units. After hatching, the nauplii were gently rinsed with tap-water, collected on a 125 μm mesh, rinsed, and offered to seahorse juveniles. The biochemical compositions of the prey are provided in [36]. The mean dry weight and length of the copepods were 1.67 ± 0.01 µg and 614 ± 140 µm, respectively, and 1.67 ± 0.04 µg and 576 ± 82 µm in *Artemia* nauplii, respectively.

The rearing was maintained until 60 DAR (days after the male’s pouch release), and the isotopic study was carried out on the periods of feeding on *Artemia* nauplii (6–60 DAR in diet A6; 11–60 DAR in diet A11).

### 2.3. Sampling and Analyses

Samples of juveniles were regularly collected to determine carbon (δ^13^C) and nitrogen (δ^15^N) isotope values, the elemental concentration of C and N, wet weight, and standard length. Samples of juveniles for stable isotope analysis (SIA) and weight and length measurements were randomly collected (*n* = 4 per diet) at 6, 11, 18, 25, 32, 46, and 60 DAR from each aquarium before the first daily feed administration. Samples of copepods and *Artemia* nauplii were also collected (*n* = 5) at different times along the experimental period, rinsed with distilled water, and kept frozen at −80 °C until further analysis.

Sampled juveniles were anaesthetized with tricaine methane-sulfonate (MS222; 0.1 g L^−1^) (Sigma-Aldrich, Darmstadt, Germany), transferred to Petri dishes, photographed, and weighed individually on a Sartorius microbalance (± 0.01 mg). Standard lengths (SL) were measured (SL = head + trunk + curved tail) from digital photographs using image processing software (NIS Elements, Nikon Tokyo, Japan).

For SIA, whole seahorses were rinsed with distilled water, frozen at −20 °C, freeze-dried, dried in oven for 24 h at 60 °C, and homogenized (Mini Beadbeater-6018 homogenizer, BioSpec, Bartlesville, USA). The analyses were made in bulk seahorses on sub-samples of about 1 mg dry weight biomass. Due to potential alterations in δ^13^C and, to a lesser extent, δ^15^N values, it is recommended that samples with a high lipid content (commonly >5% weight or C:N > 3.56) [40] be defatted for SIA [41]. Since C:N values were higher than 5% in some samples (i.e., especially in the prey), we applied specific internal conversion factors for lipid normalization (0.940, 0.922 and 0.903 for δ^13^C in copepods, *Artemia* nauplii, and *H. reidi* juveniles, respectively; 1.370, 1.059, and 1.019 for δ^15^N in copepods, *Artemia* nauplii, and *H. reidi* juveniles, respectively)

δ^13^C and δ^15^N values and elemental composition (total C and N percentage) were analyzed at Servizos de Apoio á Investigación (SAI) of the University of A Coruña (Spain). Samples were measured by continuous-flow isotope ratio mass spectrometry using a FlashEA1112 elemental analyser (Thermo Finnigan, Monza, Italy) coupled to a Delta Plus mass spectrometer (FinniganMat, Bremen, Germany) through a Conflo II interface. Carbon and nitrogen stable isotope abundance was expressed as per mil (‰) relative to VPDB (Vienna Pee Dee Belemnite) and Atmospheric Air, according to the following equation:

*δ*X = (R_sample_/R_reference_) − 1,

where X is ^13^C or ^15^N and R is the corresponding ratio of ^13^C/^12^C or ^15^N/^14^N. As part of an analytical batch run, a set of international reference materials for δ^15^N values (IAEA-N-1, IAEA-N-2, USGS25) and δ^13^C values (NBS 22, IAEA-CH-6, USGS24) were analyzed. The range of C:N ratios in sampled tissues (2.8–5.7) were within the range (0.4–6.9) of the reference materials used. The precision (standard deviation) for the analysis of δ^13^C and δ^15^N of the laboratory standard (acetanilide) was ± 0.15‰ (1-sigma, *n* = 10). Standards were run every 10 biological samples. The SIA procedure fulfils the requirements of the ISO 9001 standard. The laboratory is submitted to annual intercalibration exercises (e.g., Forensic isotope ratio mass spectrometry scheme–FIRMS, LGC Standards, Lancashire, UK).

### 2.4. Data Treatment

For comparative purposes among treatments, changes in *δ*^13^C and *δ*^15^N were studied by modelling the period comprising days 6 (diet A6) or 11 (diet A11) and 60, when only *Artemia* nauplii were offered. Isotopic data from seahorse juveniles were described applying two first-order one-compartment models [42] as functions of growth (relative dry weight increase) or development progress (days) [43,44]:

- Growth-based model G [21]

The empirical equation that describes the isotopic changes occurring during growth is as follows:

*δ* = *δ*_eq_ + *a* W_R_*^c^*

where *δ*_eq_ is the model-fitted *δ*^15^N or *δ*^13^C isotopic ratio in equilibrium with the diet. W_R_ is the ratio between the weight attained at sampling times (W_t_) and the weight when the food was switched (W_i_), *c* is the metabolic decay constant indicative of the relative contribution of metabolic turnover to changes in isotopic ratios, and *a* is a constant provided by model-fitting. The value of *a* is the difference between the initial isotopic value (when the food was switched) and the equilibrium isotopic value (*a* = δ_i_ − δ_eq_)*. δ* corresponds to the isotopic (*δ*^13^C or *δ*^15^N) value at weight W_t_.

When c = −1, turnover is due to growth only (simple dilution model), whereas c-values < −1 indicate greater proportional contributions of metabolic turnover to overall isotopic shift, with more negative values representing greater contributions of metabolic turnover [21].

- Development-based model D (adapted from [23])

Changes in stable isotope ratios were modelled as an exponential function of development progress. The model is represented as follows:

δ = δ_eq_ + *a* e^−(*m*+*k*) t^

where δ, *δ*_eq_, and *a* are as previously defined in Model G and t is the time (days) of feeding on the experimental diet, *m* is the model-fitted metabolic constant, and *k* is the growth rate parameter calculated for each duplicate considering dry weight changes from the day of diet shift (days 6 or 11) to the final experimental day (day 60). The growth rates *k* were calculated at each sampling day as:

*k* = ln(W_R_)/t

As in most studies using the model-fitting from [23] equation (model D), we assumed that growth and metabolism interact independently even though it is known that body size and metabolism covariate [45,46].

To determine half-life (G_50_ or D_50_) or equilibrium (G_95_ or D_95_) tissue turnover, the equations were solved for α = 50% or 95%, respectively. The x-fold increase in dry weight (G_α_) and the days (D_α_) required to attain a given percentage tissue turnover were calculated as:

G_α_ = e^ln (1−α/100)/*c*^ (Model G),

see [47]

D_α_ = ln (1 − α/100)/(*m* + *k*) (Model D),

see [48].

*k*, G_α_, and D_α_ values were calculated for each experimental group.

The relative contribution of tissue turnover derived from growth (P_g_) and metabolism (P_m_) was calculated as follows:

P_g_ = 2(G_50_ − 1)/G_50_

P_m_ = (2 − G_50_)/G_50_

Diet–tissue discrimination factors (Δδ) for *δ*^13^C and *δ*^15^N were estimated as the difference between the fish tissue in equilibrium and diet (Δδ = *δ*X_eq_ − *δ*X_diet_) [42].

Values are provided as mean ± standard deviation. A Shapiro–Wilk test was used to test for the normality of variables. Analysis of variance (ANOVA Univariate General Linear Model) was applied to estimate the effects of diet on survival, growth parameters, and isotope data. When significant differences were found at an alpha value of 0.05, Tukey’s HSD post-hoc test was applied to determine the significance of pairwise differences. Statistica 8.0 (StatSoft, Tulsa, OK, USA) software was used to perform statistical analyses and model-fitting.

### 2.5. Bioethics

Animal maintenance and manipulation practices were conducted in compliance with all bioethics standards on animal experimentation of the Spanish Government (Real Decreto 1201/2005, 10th October) and the Regional Government Xunta de Galicia (REGA ES360570202001/15/FUN/BIOL.AN/MPO01 and ES360570202001/16/EDU-FOR07/MPO01).

## 3. Results

### 3.1. Survival and Growth

**Figure 1:**
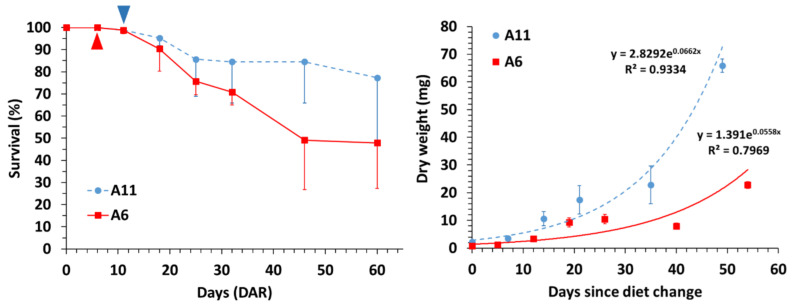
Survival (%) and dry weight (mg) in *Hippocampus reidi* juveniles fed on dietary schedules A6 and A11. Data are provided as means (two batches per diet) and standard deviations (vertical bars). Triangles: dietary shift (from copepods to *Artemia* nauplii) in A6 and A11.

Mortalities in juveniles occurred from DAR onwards in both treatments and a high mortality (21.7%) was noticed from 32 DAR to 46 DAR in diet A6 (Table 1; Figure 1). The highest final survival was attained with diet A11 (77.4 ± 28.6%), but it did not differ significantly from that in diet A6 (47.9 ± 20.6%) due to the large standard deviations (Tukey HSD test; *p* = 0.723).

Growth rates (G_R_; mg day^−1^) in seahorse juveniles were estimated from diet shifting (11 DAR in diet A1; 6 DAR in diet A6) to the end of the experimental period (60 DAR) (Figure 1). Final dry weights and final standard lengths for diet A11 (65.85 ± 7.41 mg; 51.21 ± 5.87%, respectively) were significantly higher than in diet A6 (22.78 ± 3.11 mg; 35.01 ± 1.80%, respectively) (Tukey HSD test; *p* < 0.0001 and *p* < 0.001, respectively) (Table 1; Figure 1).

C:N ratios declined significantly from 6 DAR to 11 DAR (ANOVA; F_(6,14)_ = 254.7, *p* < 0.001) and subsequently decreased slightly but not significantly until 60 DAR (2.85 ± 0.04 in A6; 2.91 ± 0.08 in A11) (Table 1). The ratios in A11 were significantly higher than in A6 (ANOVA; F_(1,14)_ = 4.95, *p* = 0.043).

### 3.2. Isotopic Variation with Ontogeny

Mean *δ*^13^C and *δ*^15^N values in copepods were −21.1 ± 0.7‰ and 5.2 ± 0.4‰, respectively. Mean *δ*^13^C and *δ*^15^N values in *Artemia* nauplii were −18.0 ± 1.9‰ *δ*^13^C and 12.3 ± 0.8‰ *δ*^15^N, respectively.

**Figure 2:**
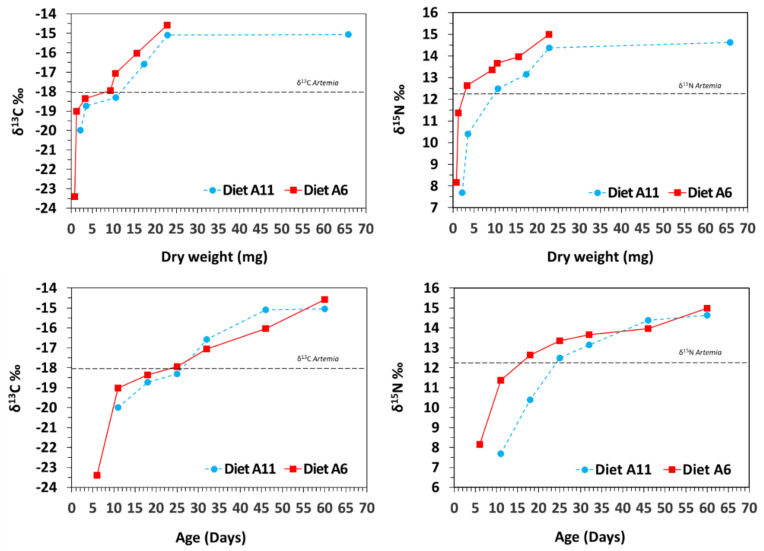
Isotopic (*δ*^13^C and *δ*^15^N) changes in seahorse *Hippocampus reidi* juveniles fed A6 and A11 diets. Data are provided as means for dry weight (mg; upper) and age (days; bellow). For clarity, sd values are not provided. Diet: nauplii of *Artemia* (dashed lines; −18.0‰ *δ*^13^C; 12.3‰ *δ*^15^N).

Isotopic values (*δ*^13^C and *δ*^15^N) increased progressively from first feeding on *Artemia* (11 DAR in A11; 6 DAR in A6) until the end of the experiment (60 DAR) (Figure 2). The shift from copepods to *Artemia* was characterised by sharp and fast enrichments in *δ*^13^C and *δ*^15^N during the first 5 days in diet A11 (1.3 and 2.7‰ increase, respectively), and especially in diet A6 (4.4 and 3.2‰, respectively). Afterwards, the enrichment was gradually reduced until reaching *δ*^13^C and *δ*^15^N final values of −15.05 and 14.63‰ for diet A11 and −14.59 and 14.98‰ for diet A6 (Figure 2). Final isotopic values in both treatments were not significantly different for *δ*^13^C (Tukey HSD test; *p* = 0.832) or for *δ*^15^N (Tukey HSD test; *p* = 0.994).

### 3.3. Turnover and Discrimination Factors

**Figure 3:**
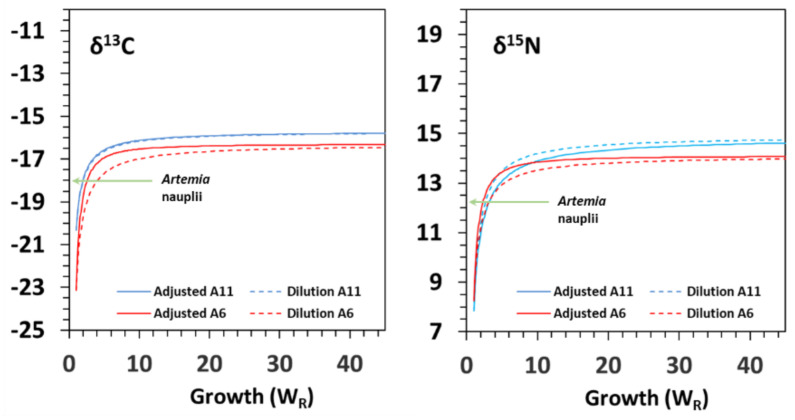
Model G–Relationships of changes in *δ*^13^C and *δ*^15^N (‰) as functions of the relative mass increase in seahorse *Hippocampus reidi* juveniles fed on diets A6 and A11. The curves refer to the feeding period on *Artemia* (6–60 DAR in diet A6 and 11–60 DAR in diet A11). Diet: *Artemia* nauplii (−18.03‰ *δ*^13^C; 12.27‰ *δ*^15^N). Dashed lines represent the dilution models.

**Figure 4:**
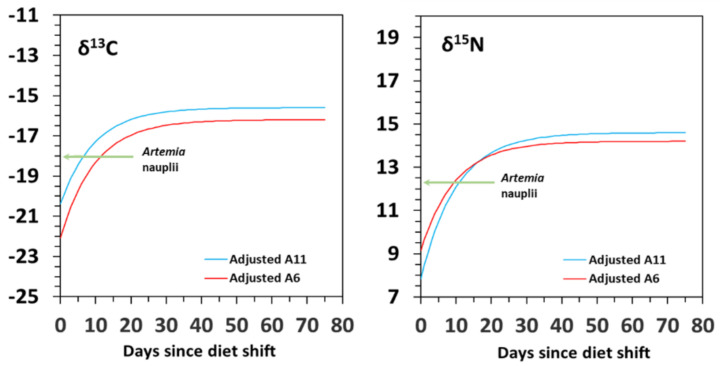
Model D–Relationships of changes in δ^13^C and δ^15^N (‰) as functions of the relative increase in days (since diet change) in seahorse *Hippocampus reidi* juveniles fed on diets A6 and A11. The curves refer to the feeding period on *Artemia* (11–60 DAR in diet A11; 6–60 DAR in diet A6). Diet: *Artemia* nauplii (−18.03‰ δ^13^C; 12.27‰ δ^15^N).

Fitting of isotopic values to growth (G) and time-based models (D) provided very high goodness-of-fit (R^2^) (Table 2; Figure 3 and Figure 4). The isotopic equilibrium in challenged juveniles was reached with higher *δ*^13^C (less negative) and *δ*^15^N values in diet A11 than in diet A6. Both fitting models performed similarly.

For comparative purposes among fitting models, G_50_ and G_95_ turnover estimates from Model G were transformed into chronological turnover rates (D_50_ and D_95_; days) (Table 2). A similar transformation was applied to convert D_50_ and D_95_ values in G_50_ and G_95_ estimates. For that, the relationship between chronological time of development and juvenile weight for each experimental batch was considered.

Regarding seahorse weight, models G and D provided similar half-life (G_50_) and near-complete turnover (G_95_) estimates (Table 2; Figure 3). Half-life G_50_ ranged from 1.6 to 2.5-fold increase in weight for *δ*^13^C and from 1.7 to 2.5- fold increase in weight for *δ*^15^N. Chronologically (model D), isotopic turnovers D_50_ and D_95_ were lower than those from model G (Table 2) for both isotopes, especially in diet A11 (Table 2; Figure 4). Half-life D_50_ ranged from 6.6 to 10.1 days for *δ*^13^C and from 6.7 to 12.4 days for *δ*^15^N.

According to model G, the isotopic equilibrium after the dietary shift in diets A6 and A11 would be reached after 36.3 and 74.4 days for *δ*^13^C and after 40.3 and 57.3 days for *δ*^15^N, respectively (Table 2). For model D, the equilibrium would be reached in about one week (W_R_ range: 8.5–9.4), independently of the isotope and diet considered.

**Figure 5:**
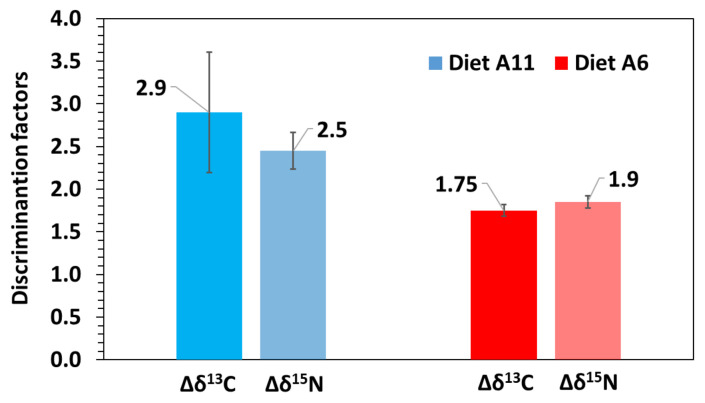
Average discrimination factors for δ^13^C and δ^15^N considering fitting models G and D in seahorse *Hippocampus reidi* juveniles fed on diets A6 (red bars) and A11 (blue bars).

Except for *δ*^13^C in model G–diet A11, the discrimination factors resulting from models D and G were rather similar for each isotope, ranging from 1.7‰ to 2.4‰ for *δ*^13^C and from 1.8‰ to 2.3‰ for *δ*^15^N (Table 2). The discrimination factor estimated for *δ*^13^C in model G–diet A11 (3.4‰) was overestimated compared to the other estimates (2.3–2.6‰). Average discrimination factors considering both fitting models were 1.8 ± 0.1‰ for *δ*^13^C and 1.9 ± 0.1‰ for *δ*^15^N in diet A6, and 2.9 ± 0.7‰ for *δ*^13^C and 2.5 ± 0.2‰ for *δ*^15^N in diet A11 (Table 2; Figure 5).

**Figure 6:**
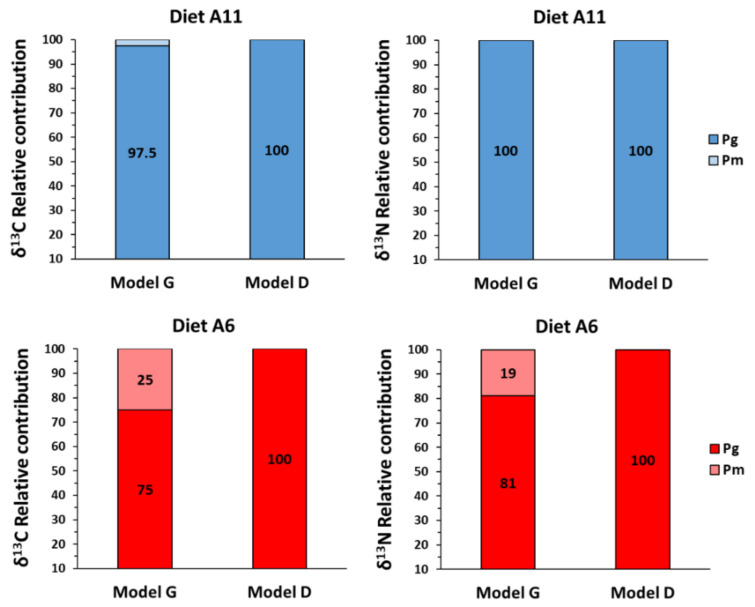
Relative contribution of growth (P_g_) and metabolic (P_m_) turnover to isotopic change (*δ*^13^C and *δ*^15^N) estimated with a growth-based model (Model G) and a development-based model (Model D) in seahorse *Hippocampus reidi* juveniles fed on diets A6 and A11.

Considering the estimates from model D of the metabolic (P_m_) and growth (P_g_) contributions, the model revealed that there was not any contribution of *δ*^13^C and *δ*^15^N to metabolism both in diet A6 and A11 (Figure 6). These results partially agreed with those in model G, but the simple dilution model revealed moderate metabolic contributions of *δ*^13^C (25%) and *δ*^15^N (19%) in diet A6.

## 4. Discussion

An extended feeding on copepods before the introduction of *Artemia* nauplii enhanced the performance in early developing seahorse *H. reidi* juveniles. Growth rates were notably affected and even though survivals did not differ significantly, they were systematically higher in juveniles fed on copepods for 10 days (diet A11) compared to those fed on that prey for only 5 days (diet A6). Those findings were partially related to both the nutritional characteristics of the experimental prey used in this study and the limited digestive capabilities in early developing juveniles.

Even though *Artemia* nauplii is one of the most used prey in the rearing of seahorse juveniles [34], it is well known that growth and survival are enhanced when copepods are included in the initial feeding [36,49,50,51]. Numerous publications refer to the high nutritional quality (e.g., n-3 HUFA and others) of copepods and their wide range in size. The characteristics of the prey used in the present study are provided in [36].

Due to the almost complete exhaustion of yolk reserves in newborn *H. reidi* [52] and the rapid adaptation to exogenous feeding, the juveniles undergo a fast initial change towards dietary isotopic values (e.g., copepods) [53]. Previous studies have shown that when organisms are provided with a new diet isotopically different from the previous diet, their tissues will eventually reflect the isotopic signature of the new diet [24,43,54]. From 6 DAR in diet A6 and from 11 DAR in diet A11 until the end of the experimental period (60 DAR), seahorse juveniles were fed on *Artemia* nauplii. The results revealed that both feeding schedules led to similar isotopic patterns with fast and continuous progression towards the dietary isotope values. In *H. reidi*, significant changes occur in gut morphology and physiology in 12–15 DAR juveniles [52] before the transition from planktonic to benthonic lifestyle (about 20 DAR) [55]. This finding agrees with those in other seahorse species, in which the development of the first intestinal loop and mucosal folding occurs at that age [56,57]. From that age, the intestinal absorption surface progressively increases [56]. Those changes should lead to better digestive efficiencies and significant enhancements in assimilation capabilities from that age onwards.

Isotope turnover rates were estimated from data fitting to models as functions of both body mass and time. The results from model D indicate that both dietary treatments performed similarly but not identically, especially for *δ*^13^C turnover (see fitted curves in Figure 4). However, inter-treatment differences were revealed by model G (body mass gain). A positive relationship between stable isotope turnover rates and body weight has previously been highlighted [44]. Moreover, the role of body size or weight change in the isotopic turnover rates of fish tissues is arguably more important than time or age. This is because fish growth rates are indeterminate and highly variable, being influenced by a range of abiotic (e.g., water temperature) and biotic (e.g., food availability) factors [44,58]. That statement was confirmed by our results (model G vs. model D), especially considering inter-treatment differences in G_95_ or D_95_ for *δ*^13^C.

The isotopic equilibrium was reached before the end of the experiment period in all cases, except for *δ*^13^C in diet A11–model G (about 85.4 days). Compared to diet A6, the equilibrium estimates (*δ*_eq_) were lower in juveniles from diet A11, likely due to differences in initial diet–tissue isotopic values at diet shift (see Figure 2). Even though there is a broad variation of turnovers rates in fish larvae and juveniles, it has been suggested that tissues are in equilibrium with the diet after four to five half-lives [22,59,60], which agrees with our results (see D_50_ vs. D_95_ in Table 2).

The present study demonstrated the influence of initial isotopic values (i.e., previous feeding history) on discrimination factors. Even though average discrimination estimates in *H. reidi* juveniles from diet A11 (2.9‰ for *δ*^13^C and 2.5‰ for *δ*^13^N) were higher than in those from diet A6 (1.8‰ for *δ*^13^C and 1.9‰ for *δ*^13^N), the estimates agree with those in other fish larvae. However, differences in discrimination factors between both dietary treatments could rely on differences in juvenile condition at the onset of the experimental periods. In adult fish, trophic discrimination factors are commonly around 1.7‰ for *δ*^13^C and 2.5‰ for *δ*^15^N [61]. In marine larvae and early post-larvae, the discrimination ranges are highly variable, ranging broadly from 0.4 to 4.1‰ for *δ*^13^C and from 0.1 to 5.3‰ for *δ*^13^N (see review [62]). In *Sciaenops ocellatus* larvae, factor estimates were 1‰ for *δ*^13^C and 1.6‰ for *δ*^15^N [24]. The discrimination factor for *δ*^15^N in reared post-flexion larvae of *Thunnus thynnus* was 0.4‰ [63], much lower than in wild-caught adults maintained in captivity (1.6‰) [64]. In *Pleuronectes americanus* larvae, discrimination factor estimates were 0–2.5‰ for *δ*^13^C and –0.3‰ at 18°C and 2.2‰ at 13°C for *δ*^15^N [26].

While models G and D differed partially in the proportions of isotopic tissue turnovers attributed to growth and metabolism, both models revealed that tissue turnovers in juveniles from diet A11 were fully related to weight gain. On the contrary, the turnover rates in juveniles from diet A6 partially directed the energy to the production of new tissues, reflecting a worse nutritional condition than those in diet A11. This statement would be the consequence of limited digestive capabilities and lower nutritional conditions in the former as a result of the advanced feeding on a non-optimal prey such as *Artemia*. The dilution of an initial carbon or nitrogen pool by the addition of newly deposited biomass would not be necessary for juveniles from diet A11.

Percentage estimates for growth turnover in juvenile seahorses were lower than those reported in the larvae/juveniles of many fish species [62] but agreed with the low isotopic contribution to metabolism reported for early developing fishes. Hesslein et al. [23] examined isotopic patterns in cultured broad whitefish (*Coregonus nasus*) juveniles in response to a dietary shift and attributed 90% of the observed isotopic changes to growth. Fry and Arnold [21] investigated *δ*^13^C shifts in juvenile brown shrimp (*Penaeus aztecus*) and reported that biomass gain was the primary cause of change in isotopic composition, although a low added effect of metabolic turnover was also detected. Reported estimates for growth contribution were 90% in red drum *Sciaenops ocellatus* [24], 61–79% in chub *Squalius cephalus,* 56–71% in roach *Rutilus rutilus*, and 42–51% in the muscle of grass carp *Ctenopharyngodon idella* juveniles [60]. However, other fish species (e.g., *Fundulus heteroclitus* juveniles) showed a high metabolic contribution to isotopic turnover [65]. A whole energy budget study carried out in the larvae of the flatfish *Scophtalmus maximus* reported a progressive increasing food absorption efficiency, accompanied by a significant decrease in the energy channelled to the metabolism (from 71 to 36%) with growth [66]. Our findings are consistent with the higher contribution of metabolism to isotopic change with a decreasing growth rate (e.g., diet A6) [21,44,67,68].

An important question arises regarding the applicability of our results to ex situ production systems: How long should copepods be offered to seahorse juveniles before switching to another prey (e.g., *Artemia* nauplii)? The availability of appropriate prey in the early life stages of seahorses is of paramount importance since one of the most critical factors in developing seahorses is the low digestion capability in early feeding juveniles [35,57], especially when fed on *Artemia* in rearing systems [47,48]. That limitation has been supported visually by direct observations of feces (occurrence of undigested *Artemia* nauplii) and physiologically by the absence of supranuclear vesicles in the intestine in juveniles fed exclusively on *Artemia* [36]. Furthermore, copepods are highly preferred to *Artemia* nauplii during the first two weeks of development [51] and can be mechanically broken into smaller pieces, improving the action of digestive enzymes [36]. The results achieved on biological indicators such as growth and mortality as well as on the contribution of growth and metabolism to tissue turnover demonstrate that extending the feeding on copepods from 6 DAR to 11 DAR enhanced the overall condition and welfare of *H. reidi* juveniles. Due to the high cost of copepod production, a co-feeding regime including copepods–*Artemia* has been proposed for large-scale ex situ production [69].

In summary, the present study provided new insights for the understanding of food assimilation and growth in early developing *Hippocampus reidi* juveniles in response to dietary shift. Our results highlighted the importance of copepods as the first prey, confirming that their feeding by seahorse juveniles should be extended as long as possible before the inclusion of *Artemia* nauplii on the feeding schedule. Longer periods of initial feeding on copepods would result in higher growth rates and survival by promoting juvenile welfare and a better nutritional condition. It can be concluded that the diet is an important factor contributing to daily variations in carbon and nitrogen stable isotopes profiles in juveniles and that the feeding history has implications on discrimination factors and to a lesser extent on isotopic turnover rates. Meanwhile, the elucidation of the role of growth and metabolism on stable isotope turnovers deserves further investigation to characterize the trophic dynamics more precisely in the species. To our knowledge, this is the first study on stable isotope turnover in seahorses, providing specific diet–tissue discrimination factors for *δ*^13^C and *δ*^13^N in seahorse juveniles.

## Figures and Tables

**Table 1:** Survival, dry weight (DW), standard length (SL), and C:N ratios during the experimental period in *Hippocampus reidi* juveniles submitted to dietary treatments A6 and A11. Data are provided as means (two batches per diet) and standard deviations (sd).

Diet	Days	Survival	DW (mg)		SL (mm)		C:N	
	(DAR)	(%)	Mean	sd	Mean	sd	Mean	sd
A6	6	100	0.80	0.02	12.33	0.32	5.75	0.00
	11	98.8	1.30	0.39	15.95	2.52	3.03	0.06
	18	90.4	3.33	0.07	20.19	0.10	3.20	0.03
	25	75.7	9.28	2.30	26.41	4.12	3.07	0.37
	32	70.9	10.50	1.66	29.32	2.34	2.88	0.04
	46	49.2	15.61	4.17	31.81	5.07	2.80	0.01
	60	47.9	22.78	3.11	35.01	1.80	2.85	0.04
A11	11	98.8	2.20	0.14	18.15	0.35	3.14	0.02
	18	95.2	3.53	0.85	22.05	2.39	3.07	0.06
	25	85.7	10.63	0.04	26.74	0.40	3.29	0.10
	32	85.4	17.38	6.29	33.82	4.66	3.14	0.20
	46	84.5	22.85	2.58	36.94	3.08	3.05	0.17
	60	77.4	65.85	7.41	51.21	5.87	2.91	0.08

**Table 2:** Parameter estimates (mean ± SE) for best-fit models for growth and time based-models predicting δ^13^C and δ^15^N values in *Hippocampus reidi* juveniles in the periods 6–60 DAR (Diet A6) and 11–60 DAR (Diet A11) (*Artemia* nauplii feeding). Turnover rates: G_50_, G_95_ (x-fold increase in biomass) and D_50_, D_95_ (Days). Δδ: Trophic discrimination factor (Δδ = δX_eq_ − δX_diet)_.

***δ*^13^C**	**Model G**	**δY_eq_**	**SE**	***c***	**SE**	**R^2^**	**G_50_**	**G_95_**	**D_50_**	**D_95_**	**Δδ**
	A6	−16.3	0.5	−1.474	0.489	0.831	1.6	7.6	8.4	36.3	1.7
	A11	−15.7	0.4	−1.037	0.103	0.776	2.0	43.7	10.1	74.4	3.4
	**Model D**	**δY_eq_**	**SE**	**m**	**SE**	**R^2^**	**G_50_**	**G_95_**	**D_50_**	**D_95_**	**Δδ**
	A6	−16.2	0.9	0.040	0.070	0.816	2.5	8.8	6.8	29.4	1.8
	A11	−15.6	0.8	0.035	0.047	0.828	2.0	8.5	6.6	28.6	2.4
**δ^15^N**	**Model G**	**δY_eq_**	**SE**	***c***	**SE**	**R^2^**	**G_50_**	**G_95_**	**D_50_**	**D_95_**	**Δδ**
	A6	14.1	0.3	−1.326	0.269	0.934	1.7	9.6	9.3	40.3	1.8
	A11	14.9	0.5	−0.844	0.176	0.959	2.3	34.8	12.4	57.3	2.6
	**Model D**	**δY_eq_**	**SE**	**m**	**SE**	**R^2^**	**G_50_**	**G_95_**	**D_50_**	**D_95_**	**Δδ**
	A6	14.2	0.5	0.041	0.018	0.916	2.5	8.6	6.7	28.9	1.9
	A11	14.6	0.1	0.030	0.008	0.944	2.0	9.4	6.9	30.1	2.3

## Data Availability

The data presented in this manuscript are available at Mendeley datasets (https://data.mendeley.com/datasets/v5vbs8dn2m/1 accessed on 10 May 2022).
